# Inventory of water masses and carbonate system from Brazilian’s northeast coast: Monitoring ocean acidification

**DOI:** 10.1371/journal.pone.0271875

**Published:** 2022-07-26

**Authors:** Carlos Augusto Ramos e Silva, Nicole Silva Caliman Monteiro, Luciana Miranda Cavalcante, Waldemar Tavares Junior, Maria Eulália Rocha Carneiro, Flavo Elano Soares de Souza, Carlos Alexandre Borges Garcia, Raimundo Nonato Damasceno, Anderson de Araújo Rocha

**Affiliations:** 1 Postgraduate Program in Oceans and Earth Dynamics, Geosciences Institute, Federal Fluminense University, Niterói, RJ, Brazil; 2 Marine Biology Department, Biology Institute, Federal Fluminense University, Niterói, RJ, Brazil; 3 Center of Studies on Water, Biomass, and Oil—NAB, Federal Fluminense University, Niterói, RJ, Brazil; 4 Research and Development Center—Leopoldo Américo Miguez de Mello (CENPES), Ilha do Fundão, Cidade Universitária, Rio de Janeiro, RJ, Brazil; 5 Agricultural School of Jundiaí, Federal University of Rio Grande do Norte, Jundiaí District, Macaíba, Rio Grande do Norte, Brazil; 6 Chemistry Department, Federal University of Sergipe, University District Professor José Aloísio de Campos, São Cristovão, SE, Brazil; Public Library of Science, UNITED KINGDOM

## Abstract

This manuscript presents an inventory of the carbonate system from the main water masses comprising the marine current system on Brazil’s northeast coast (South Atlantic Ocean). For this purpose, four transects were conducted with an approximate length of 357 km (each one) through the platform and continental slope of the Sergipe–Alagoas sedimentary basin. Water samples were then collected in vertical profiles measuring from 5 to 1,799 meters depth, totaling 34 stations. Total alkalinity, calcium, and total boron were obtained analytically from these samples and by relationships with salinity. Speciation and concentration of the carbonate system were obtained by means of thermodynamic modeling. The results revealed that the empirical models used to calculate the concentrations of TA, calcium and total boron showed relevant variation when compared to the analytical values (TA: 5.0–6.5%; Ca: 0.4–4.8%; B_T_: 7.0–18.9%). However, the speciation and concentration of the carbonate system (CA, DIC, $CO_{3}^{2-}$, CO_2(aq)_, Ω_Calc_, and Ω_Arag_) obtained from the empirical values of TA, calcium and total boron did not differ significantly from those obtained analytically (0.0–6.1%). On the other hand, the parameters of pH, $HCO_{3}^{‐}$, $CO_{3(\mathrm{aq})}^{2‐}$, CO_2(aq)_, ρCO_2_, Ω_Calc_, and Ω_Arag_ varied significantly within the different water masses (p < 0.05). This study supports and encourages acidification monitoring projects in the South Atlantic Ocean, based on modeling the carbonate system parameters generated in real-time.

## Introduction

Carbon dioxide (CO_2_) sources, transport mechanisms, and transformations are an important matter in oceanography field studies [1]. CO_2_ can exhibit significant spatial and temporal variability within the same water mass since the ocean content is dependent on processes such as the atmospheric exchange through sea surface and the degradation of organic matter (both autochthonous and allochthonous derived) [2]. Increasing CO_2_ content will lead to both reduction in both carbonate content and pH ($CO_{3}^{2-}+H_{2}O+CO_{2}=2HCO_{3}^{-}$); this process is called *ocean acidification* (OA) [3]. It is known that OA can reduce the capacity of marine organisms (*e*.*g*., coccolithophorids, corals, foraminifera, and bony fishes) because of the increased concentration of H^+^ ions in seawater [4]. Understanding the acidification process is necessary for predicting future climate changes and responses from marine biota [5–7].

Efforts have been made to implement coordinated monitoring programs and increase understanding of OA [8, 9]. The main actions required for the implementation of these programs consist of (1) creating a database of the carbonate system of coastal ecosystems (spatial and temporal), (2) standardization in pH and TA determinations, and (3) transparency in precision of the polynomials responsible for generating the data of the carbonate system [8–12].

An OA monitoring network requires constant maintenance of the records of the main chemical parameters, for example pH and total alkalinity, which allows the determination of the saturation state of the aragonite (Ω_Arag_) and a complete description of the carbonate system [9]. The parameters suitable for this purpose may be defined by the balance of few reactions (Eqs 1, 2, 3, 4, and 5) that occur when CO_2_ dissolves in sea water [13–15] as shown below:

$${CO}_{2(g)}={CO}_{2(aq)},$$ (1)

$${CO}_{2(aq)}+H_{2}O_{\left(l\right)}=H_{2}{CO}_{3(aq)},$$ (2)

$$H_{2}{CO}_{3(aq)}=H_{\left(aq\right)}^{+}+{HCO}_{3(aq)}^{-},$$ (3)

$${HCO}_{3(aq)}^{-}=H_{\left(aq\right)}^{+}+{CO}_{3(aq)}^{2-},$$ (4)

$${CO}_{3(aq)}^{2-}+{Ca}_{\left(aq\right)}^{2+}={CaCO}_{3(s)},$$ (5)

Ocean acidification data (pH, TA, $\left[\mathrm{HC}O_{3}^{-}\right]$ [H_2_CO_3(aq)_] [CO_2(aq)_] ρCO_2_, Ω_Calc_, and Ω_Arag_) are also indispensable for validating CO_2_ modeling tests on both a global and regional scale.

Studies on water masses in northeastern Brazil are scarce, but some previous studies in the South Atlantic indeed covered the surface (0–200 m), intermediate (200–1000 m), and deep (below 1000 m depth) waters. Reid [16], estimated the general circulation pattern of the South Atlantic from the characteristics of the geostrophic shear, reporting contributions from North Atlantic waters. Some authors [16–21] investigated the physical-chemical characteristics (temperature, salinity, and oxygen) of the different water masses in the South Atlantic, disregarding the chemistry of the carbonate system. Apart from these works are those by Silveira [20], Campos et al. [22], and Silva [19] in the southeastern region of Brazil, but these works did not deal with the carbonate system. By contrast, Bates [23], studied the distribution of carbonate chemistry in samples collected from many water masses in the Southeast Pacific as part of the US GEOTRACES project, compared with the carbonate chemistry from samples collected 20 years earlier in the same area. Also, several recent studies with the carbonate system have been carried out in coastal waters on the Brazilian continental shelf in northeast-south-southeast regions [1, 13, 14, 22–26].

The present study aimed to compile, in an unprecedented way, an inventory of the carbonate system in water masses of the northeast margin of Brazil (Tropical Water, South Atlantic Central Water, Antarctic Intermediate Water, and Upper North Atlantic Deep Water) and to elucidate the physical-chemicals processes governing these concentrations and speciation. In addition, to verify the dynamics of the carbonate system parameters through the program Marine Chemical Analysis (AQM). This experiment provides information and encourages ocean acidification monitoring projects.

## Materials and method

### Study area

**Fig 1:**
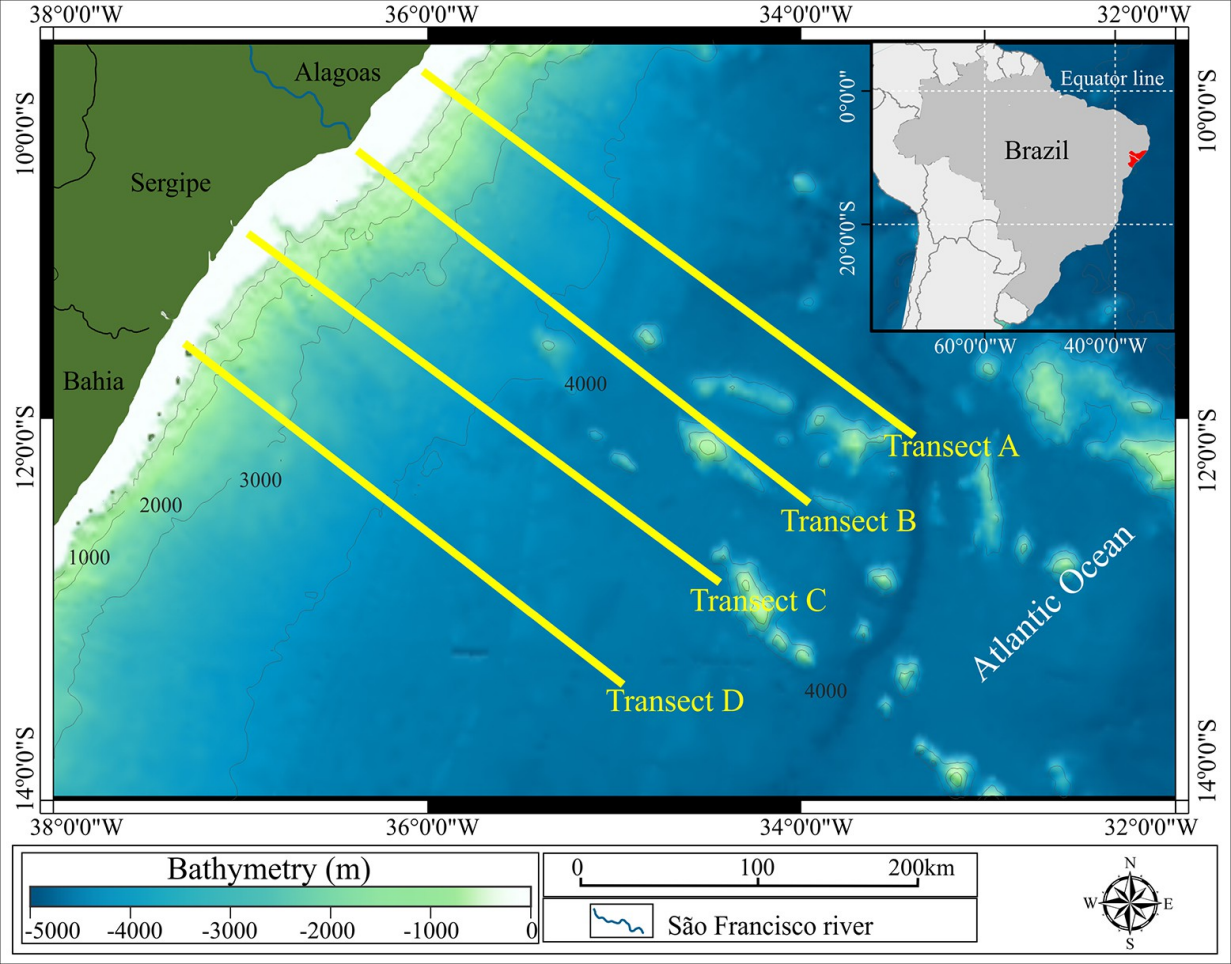
Reference map of the study site. Northeastern continental margin of Brazil, within the Sergipe–Alagoas sedimentary basin. Blue shades, local depth; yellow lines, sampling transects A, B, C, and D; blue line, São Francisco river; dark green filled shape, Brazilian states that are adjacent to transects.

The study area is located on the northeastern continental margin of Brazil, in the sedimentary Sergipe/Alagoas (SEAL) basin. The SEAL basin is divided between terrestrial and maritime domains, comprising an oil province in an advanced exploratory stage that gives economic and environmental importance to the vicinity [25]. The terrestrial domain of the basin comprises the states of Sergipe and Alagoas, which are separated by the course of the São Francisco River (Fig 1). The maritime part of the SEAL occurs along the continental shelf, which is 20 to 50 km long, and the platform slope, which occurs between 40 and 80 m deep [25]. In this context, the present study covered an extension of 350 km from the coastline, where the Tropical Water and Coastal Water are the predominant water masses [26].

### Data acquisition

The samples were collected between May 10 and 21, 2014, which is the beginning of the rainy season (127 mm^3^/month) [27]. The sampling effort took place onboard the R/V Seward Johnson during a cruise through the SEAL basin, which was made possible by financial resources and logistical support provided by the MARSEAL Project (PETROBRAS/CENPES). Collection of the samples was conducted through a CTD (conductivity, temperature, and depth sampler) coupled to a *Rosette* with capacity for 24 oceanic bottles of the Niskin and Go-Flo type (General Oceanics brand).

The collections were conducted along four transects (A, B, C, and D, Fig 1), arranged latitudinally. In each transect, eight to nine stations were established, totaling 34 profiles, with a maximum distance of 365 km from the shoreline (Table 1).

**Table 1:** Profiling stations by transect A, B, C, and D. Station: Identification number; Dist: Distance from the shoreline to each station (km).

A		B		C		D	
Station	Dist	Station	Dist	Station	Dist	Station	Dist
1	8	38	4	39	6	75	12
2	21	37	8	40	14	74	19
3	25	36	12	41	25	73	23
4	30	35	19	42	29	72	30
8	83	33	34	43	36	68	68
10	127	29	86	47	81	66	97
14	247	27	116	49	111	62	216
18	365	23	219	53	230	58	335
		19	340	57	349		

Sampling depths were established from six isobaths, *i*.*e*., 5, 20, 250, 700, 1250, and 1650 m, that correspond to the current water mass’s system in the South Atlantic (TW, Tropical Water; SACW, South Atlantic Central Water; AAIW, Antarctic Intermediate Water; and UNADW, Upper North Atlantic Deep Water), according to Silveira [20]. The boundaries within each water layer were determined from the CTD data (Table 2), which were also used to conduct the T-S (temperature–salinity) diagram.

**Table 2:** Fieldwork recognition of the water masses (Wm). Thermohaline limits, sampling depths, and referential depths [20].

Wm	Temperature (°C)	Salinity (g/kg)	Sampling depth (m)	Wm depths (m) [31]
**MW** ^**1**^	27.91	35.56	4.3	–
**TW** ^**2**^	27.37–28.26	36.44–37.55	4–21	0–142
**SACW** ^**3**^	13.33–15.59	35.41–35.78	202–299	142–567
**AAIW** ^**4**^	4.12–5.59	34.53–34.93	598–1250	567–1060
**AAIW/UNADW**	3.89–4.37	34.70–35.13	999–1650	1060–1300
**UNADW** ^**5**^	3.56–4.28	35.09–35.13	1398–1899	1300–3260

^1^Mixture Water.

^2^Tropical Water.

^3^South Atlantic Central Water.

^4^Antarctic Intermediate Water.

^5^Upper North Atlantic Deep Water.

**Fig 2:**
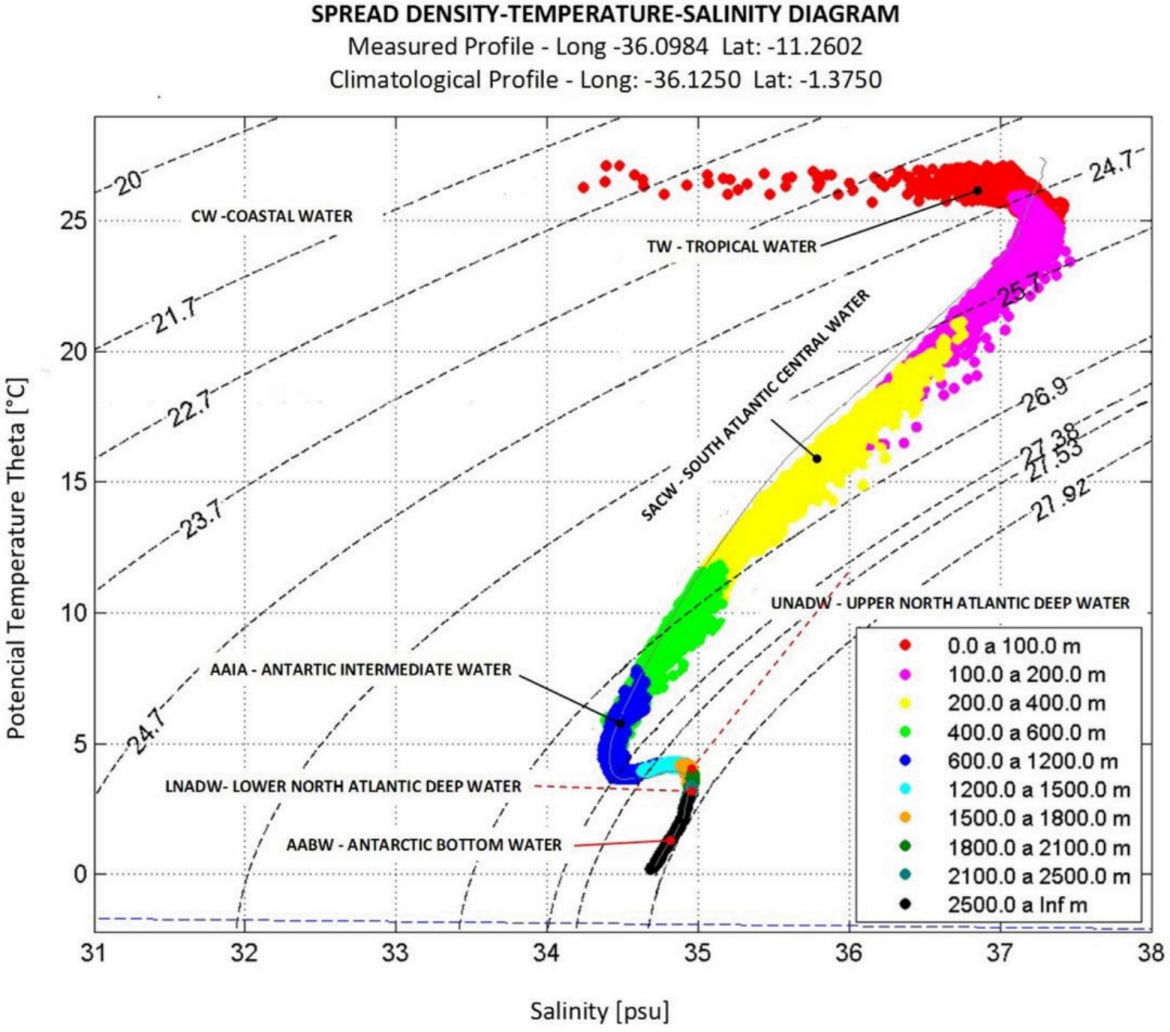
T-S diagram indicating the distinct water masses from the studied area (maritime sedimentary SEAL basin).

For each collection station, different sampling depths were defined according to the CTD information (T-S diagram), allowing for the identification of the interfaces between the different water masses (Fig 2); further information can be found on the S1 Table.

### Thermodynamic modeling

The modeling and the procedural calibrations described in this manuscript were performed with the Marine Chemical Analysis (AQM) program [1]. The AQM is a package of thermodynamic equations, executed via MS Excel, which can predict the complex composition of the marine carbonate system based on measurements that can be made relatively inexpensively such as pH, temperature, and alkalinity reducing the overall costs of ocean acidification monitoring programs. The AQM program is available upon request to the corresponding author’s email.

### Statistical procedure

Data normality was verified using the Shapiro-Wilks test, and based on this result, the non-parametric Kruskal Wallis test was chosen for comparisons between groups. All statistical tests were performed using the Statistica 7.0 software (TIBCO) with a significance level set at p<0.05.

### Analytical procedure

The analytical method was based on international procedures for studies involving the chemistry of inorganic carbon dioxide in marine waters [11, 28–32] with the necessary adaptations (S2 Table).

### T-S diagram

Temperature and salinity data from CTD profiling were utilized to make the profiles and vertical sections of temperature, salinity, and potential density of the stations using the T-S diagram. The water masses were aimed to be determined by mapping their interfaces, characterizing their core, and isopycnals. The optimal multiparametric analysis was utilized to provide data on the interface limits and at the average core depth of each water mass. This allowed locating each layer in the T-S diagram parameterized by the depth range associated with its typical values of temperature, salinity, and potential density [33, 34]. The density of the water masses was displayed as isopycnals on the T-S diagram.

#### Absolute salinity (S_A_)

The S_A_ (g/kg) was calculated from the electrical conductivity measured *in situ* (CTD) and the thermodynamic equation of seawater [35]. Absolute salinity is the most accurate parameter established to estimate the real salinity of the environment. Unlike other scales (for example, conductivity and practical salinity), S_A_ also considers the non-electrolytic salts through algorithms extracted from the data on the water’s chemical composition in a given region [35].

#### TA

For the determination of TA, water samples were collected and filtered in a Nalgene filtration system through GF/F filters before being transferred to BOD type flasks (300 mL, from the Kimble brand) and immediately analyzed [28].

The potentiometric determination was conducted with duplicate samples in an open thermostated glass cell, where 3 mL (to obtain v1) and 10 mL (to obtain v2) of HCl 0.1 M were added to each 100 mL sample [36]. The method consists in determining the slope of the line by obtaining two points for the function of Gran (F): F (1) defined by v1 and F (2) defined by v2 [36]. A Thermo Scientific Orion Star potentiometer coupled to the Orion glass reference electrode cell, model 8102BNUWP was used for potentiometric determinations. The pH electrode was calibrated daily with "Tris" buffer (0.04 m) for sample readings (maximum 15 samples per day). Due to the reduced number of samples per day, the short period of the oceanographic cruise, and the constant working conditions (electricity source, solutions, and equipment), we chose to verify the electrode performance at the beginning and the end of the oceanographic cruise. The electrode’s percent efficiency ranged between 99.49% and 99.54% concerning the theoretical Nernst value (59 mV). More details are available in hydrogen potential (pH).

The analytical precision and accuracy were calculated from five replicates of the reference material (Dickson–CRM, for oceanic CO₂ measurements, batch 104) [37], which obtained a 95% recovery rate from the expected value (Table 3). The calculated TA was obtained by the AQM program through the equation (TA (μmol/kg) = 660 + 47.6S) defined by Hunter [38] for waters of the Atlantic and Pacific oceans by the GEOSECS Program. The normalized total alkalinity (NTA) was obtained by the AQM program using the equation (NTA (μmol/kg) = TA (μmol/kg)x35/Salinity (g/kg), 35 was assumed to be the representative salinity of the water masses.

**Table 3:** Total alkalinity measured from 5 replicates of the certified reference material (Dickson, oceanic CO_2_ measurements, batch 134).

	Expected value	Measured value	Accuracy		Precision	Sample volume
		Average (n = 5)	Absolute error	Relative error	Variation coefficient	mL
**Total alkalinity**	2222.6 μmol/kg	2108.0 μmol/kg	−115.0	−5.0	1.66%	50

#### Hydrogen potential (pH)

The total pH of the water samples collected during the cruise was determined in the R/V Seward Johnson "wet laboratory" as follows: pH_T_ (= -log([H^+^] + [(HSO_4_^-^]/c^o^), where c^o^ is the thermodynamic concentration (1 mol/kg-soln).

The internal solution of the combined pH electrode was filled up with 0.7 m NaCl to reduce the potential liquid junction. The electromotive force (emf) of the electrode was related to the molar concentration of the proton [H^+^], as shown in Eq 6.

$$E=E^{o}-\left(\frac{RT}{F}\right)\ln\left[H^{+}\right],$$ (6)

where E° is the standard electrode potential, which was determined by titrating a 0.7 m NaCl solution with 0.179 M HCl [29]. The pH_T_ (total scale) values were measured immediately after each collection at a constant temperature of 25°C in a thermostatic cell connected to a microprocessed thermostatic bath with external circulation (Qimis) to avoid temperature bias [39]. The determinations were made by the Thermo Scientific Orion Star potentiometer coupled to the Orion glass reference electrode with a 0.7 m NaCl outer chamber filling solution, model 8102BNUWP. The analytical slope for the electrode was within ± 0.13 mV (teoretical Nersnt value at 25°C). The electrode was calibrated with a "Tris" buffer (0.04 m) prepared in the laboratory [40], where pH values were assigned by spectrophotometry (m-cresol method) [14, 28, 41]. The "Tris" buffer allows accuracy of 0.001 pH units [40, 42]. Subsequently, using the AQM program, the pH results were corrected for the temperature recorded at the sampling moment (pH_t_ = pH_25_ + A + Bt + Ct^2^) [43].

#### Calcium (Ca) and total boron (B_T_)

The determination of Ca and B_T_ was conducted using a MIP OES (microwave-induced plasma optical emission spectrometer, 4200 MP-AES, Agilent brand). The external analytical curves were made with monoelementary standards (1000 mg/L, VHG®) with concentrations in the range of 0.1 to 10 mg/L, in an ultrapure water matrix. A matrix influence test was conducted in which it was found that both the boron and calcium signals did not show any significant difference between the ultrapure water matrices and the 500 mg/L NaCl solution. The calculated calcium and boric acid were also obtained using the equations described by Millero, respectively [44, 45]; [Ca^2+^]_T_ = 2.938x10^-4^xS, [B]_T_ = 0.000416x(S/35).

#### CO_2_ inorganic system

All parameters from the inorganic CO_2_ system (CO_2_, ${\mathrm{CO}}_{3}^{2‐}$, ${\mathrm{HCO}}_{3}^{‐}$, DIC, ρCO_2_, Ω_Calc,_ and Ω_Arag_) were calculated using the carbonate system dissociation constant *K* [46] defined as follows:

- ${lnk}_{B}^{*}$ [37]
- ${lnk}_{Si}^{*}$ [45]
- ${lnk}_{1}^{*}$ (H_3_PO_4_) [47]
- ${lnk}_{2}^{*}$ ($H_{2}{PO}_{4}^{-}$) [47]
- ${lnk}_{3}^{*}$ (${HPO}_{4}^{2-}$) [47], and
- ${lnk}_{2}^{*}$ (${CO}_{3}^{2-}$) [48].

The aqueous concentrations (CO_2(aq)_) and the partial pressure (ρCO_2_) were calculated from the variables of temperature, salinity, pH, and TA and by using the thermodynamic and stoichiometric constant *K* (${pk}_{1}^{o}$, ${pk}_{2}^{o}$, ${pk}_{1}^{*}$, and ${pk}_{2}^{*}$) [10, 49]. The AQM was also used in this phase, aiding the calculations.

## Results and discussion

### T-S diagram

The identification of water masses was conducted via the T-S diagram (Fig 2) methodology, which uses the temperature and salinity data [50] to obtain the depth boundaries of water masses.

In this way, an existing variation in our temperature and salinity values could be perceived with those of the other authors mentioned in Table 4. Furthermore, the Upper Circumpolar Deep Water was not addressed in this work because of the complexity of its definition, such as the oxygen and nutrients concentrations, to differentiate this water mass with the overlying (AAIW) and underlying (UNADW) masses [20].

**Table 4:** Temperature (°C) and salinity (g/kg) for each water mass (Wm). Comparison with previously published results.

Wm	Temperature (°C)	Salinity (g/kg)	Reference
**TW**	27.37–28.36	36.44–37.55	**The present study**
	>20.00	>36.17	[51]
	>20.00	>36.37	[20]
	>18.00	>36.17	[19]
	20.00–27.00	–	[52]
**SACW**	13.33–15.59	35.42–35.78	**The present study**
	5. 00–20.00	34.46–36.17	[52]
	8.72–20.00	34.82–36.37	[20]
	6.00–20.00	34.76–36.17	[53]
	10.00–20.00	35.16–36.17	[51]
	–	34.81–36.17	[54]
	5.95–18.35	34.68–36.57	[19]
**AAIW**	4.12–5.59	34.53–34.93	**The present study**
	3.46–8.72	34.58–34.82	[20]
	4.92–5.90	34.64–34.94	[19]
**NADW**	3.56–4.37	35.09–35.13	**The present study**
	3.00–4.00	34.76–35.16	[53]
	2.04–3.31	34.75–35.03	[20]

The salinity from the superficial layer (4.3 m deep) of station 36 was lower than the expected value (>36.37 g/kg) for a TW mass [20]. A continental freshwater input may explain this difference; thus, this sample was called “Mixture Water” (MW).

### Measured versus predicted values

**Calcium and total boron.** The values analyzed and calculated in the different water masses varied below 5% for calcium (Ca^2+^) and between 7.03% and 18.94% for total boron (B_T_) (Table 5).

**Table 5:** Calcium and total boron concentrations (μmol/kg; mean ± standard deviation) in water samples from 34 stations. Analytically obtained levels: microwave-induced plasma atomic emission spectrometry. Predicted concentrations: calculated using the AQM program using the absolute salinity scale (g/kg). When calculating the relative error (RE%), the expected value is the analyzed value.

Wm	Sample Sizes	Ca^2+^_analyzed_	Ca^2+^_calculated_	RE (%)	B_T analyzed_	B_T calculated_	RE (%)
**MW**	*n = 1	10180	10664	**4.75**	357	410	**14.91**
**TW**	*n = 63	11168 ± 199	11210 ± 63	**0.38**	383 ± 23	410 ± 0.04	**7.03**
**SACW**	*n = 24	10570 ± 221	10716 ± 33	**1.39**	362 ± 26	412 ± 0.08	**13.77**
**AAIW**	*n = 22	10203 ± 163	10417 ± 26	**2.10**	347 ± 26	412 ± 0.02	**18.94**
**AAIW/UNADW**	*n = 18	10287 ± 154	10540 ± 28	**2.46**	353 ± 36	412 ± 0.02	**16.86**
**UNADW**	*n = 15	10303 ± 227	10587 ± 3	**2.76**	352 ± 36	412 ± 0.01	**16.99**

RE = relative error.

In this validation step, the calculated (AQM) concentrations for calcium and boron emphasized that these values may be recommended instead of those obtained analytically to investigate the carbonate system involving the same water masses. Calcium concentration is important for the equations that determine the saturation state of calcite. The concentration of boron allows obtaining, through equations, the concentrations of carbonate alkalinity [CO_2_], [${\mathrm{CO}}_{3}^{2‐}$], [${\mathrm{HCO}}_{3}^{‐}$], and DIC [10, 28]. The variation presented for Ca (analyzed and calculated) did not show relevant interference in the predicted values for the carbonate system (Table 6). Regarding the total estimated boron, despite having a variation of 18.94%, relative to the analyzed value, we also recommend its application for studies of the carbonate system in these water masses, since the concentrations obtained through the calculated B_T_ showed tiny variation (<1%) (Table 6).

**Table 6:** Variations in the carbonate system parameters as a function of the minimum (a, c) and maximum (b, d) values found for Ca^2+^ and B_T_ (Table 5). Values obtained with the AQM program keeping the temperature (25°C), salinity (35 g/kg), pH_**T**_ (8.0), and TA (2300 μmol/kg) variables constant at the program entry. The expected value is the analyzed value.

	CA μmol/kg	Ω_Calc_	Ω_Arag_	HCO_3_^-^ μmol/kg	CO_3_^2−^ μmol/kg	DIC μmol/kg	CO_2_ μmol/kg
**Ca**^**2+**^_**(a)**_	2173	5.0	3.3	1704	210	1921	12.0
**Ca**^**2+**^_**(b)**_	2173	5.2	3.5	1704	210	1921	12.0
**Δ%**_**(b-a)**_	**0.0**	**4.0**	**6.1**	**0.0**	**0.00**	**0.0**	**0.0**
**B**_**T (c)**_	2173	5.0	3.3	1704	210	1931	12.0
**B**_**T (d)**_	2160	5.0	3.3	1694	209	1914	12.0
**Δ%**_**(d-c)**_	**-0.6**	**0.0**	**0.0**	**-0.6**	**-0.5**	**−0.86**	**0.0**

(a) 10180 μmol/L (analyzed), (b) 10664 μmol/L (calculated), represent the largest RE (%) in the Table 5. (c) 347 μmol/L (analyzed); (d) 412 μmol/L (calculated). CA = carbonate alkalinity (TA-ΣBi; Bi = bases). The analyzed and calculated values were obtained from Table 5, with the highest RE (%).

#### Practical versus absolute salinity

The S_A_ (g/kg) has been considered the most accurate measure to estimate the real salinity of the environment [35]. However, practical salinity unit (psu) has been widely used by researchers to generate carbonate system values [1, 6, 14, 15, 55]. Thus, the AQM program was fed with both the psu and g/kg salinity scales to test the existence of relevant interference when predicting the carbonate system fractions (obtained from the analytical TA) from the studied water masses (Table 7).

**Table 7:** Effects of the practical (psu) and absolute salinity (g/kg) scales when generating the carbonate system parameters. Values obtained from calculated total alkalinity with the AQM program [1, 13–15]. Further information can be found in the methodology section. In calculating the relative error (RE%), the parameters obtained by the absolute salinity (g/kg) are the expected values.

Parameters	Water mass					
	MW	TW	SACW	AAIW	AAIW/UNADW	UNADW
**Ca**^**2+**^ **(psu)**	10613	11156 ± 63	10665 ± 33	10365 ± 26	10488 ± 28	10536 ± 3
**Ca**^**2+**^ **(g/kg)**	10664	11210 ± 63	10716 ± 33	10417 ± 26	10540 ± 28	10587 ± 3
**RE (%)**	**-0.5**	**-0.5**	**-0.5**	**-0.5**	**-0.5**	**-0.5**
**B**_**T**_ **(psu)**	410	410 ± 0.0	412 ± 0.1	412 ± 0.0	412 ± 0.0	412 ± 0.0
**B**_**T**_ **(g/kg)**	410	410 ± 0.0	412 ± 0.1	412 ± 0.0	412 ± 0.0	412 ± 0.0
**RE (%)**	**0.00**	**0.00**	**0.00**	**0.00**	**0.00**	**0.00**
**TA (psu)**	2345	2429 ± 9.7	2347 ± 5.4	2298 ± 3.9	2317 ± 4.4	2324 ± 0.4
**TA (g/kg)**	2352	2437 ± 9.7	2355 ± 5.4	2306 ± 4.0	2325 ± 4.4	2332 ± 0.4
**RE (%)**	**-0.3**	**-0.3**	**-0.3**	**-0.3**	**-0.3**	**-0.3**
**NTA (psu)**	2242	2194 ± 42	2235 ± 11	2280 ± 9	2263 ± 11	2262 ± 19
**NTA (g/kg)**	2232	2184 ± 42	2224 ± 11	2269 ± 9	2252 ± 11	2251 ± 19
**RE (%)**	**4.3**	**0.5**	**0.5**	**0.5**	**0.5**	**0.5**
**Ω**_**Calc**_ **(psu)**	5.47	5.78 ± 0.27	3.30 ± 0.26	1.95 ± 0.28	1.72 ± 0.16	1.72 ± 0.13
**Ω**_**Calc**_ **(g/kg)**	5.42	5.81 ± 0.26	3.27 ± 0.26	1.93 ± 0.28	1.71 ± 0.16	1.70 ± 0.12
**RE (%)**	**0.9**	**-0.5**	**0.9**	**1.0**	**0.6**	**1.2**
**Ω**_**Arag**_ **(psu)**	3.64	3.85 ± 0.18	2.12 ± 0.17	1.23 ± 0.18	1.08 ± 0.10	1.08 ± 0.08
**Ω**_**Arag**_ **(g/kg)**	3.61	3.82 ± 0.18	2.10 ± 0.17	1.22 ± 0.18	1.08 ± 0.10	1.08 ± 0.08
**RE (%)**	**0.8**	**0.8**	**1.0**	**0.8**	**0.0**	**0.0**
**CO**_**3**_^**2-**^ **(psu)**	231.18	238 ± 11	137 ± 10	81 ± 12	71 ± 6	72 ± 5
**CO**_**3**_^**2-**^ **(g/kg)**	231.69	238 ± 11	136 ± 10	81 ± 12	71 ± 6	72 ± 5
**RE (%)**	**−0.2**	**0.0**	**0.7**	**0.0**	**0.0**	**0.0**
**DIC (psu)**	1873	1822 ± 43	2019 ± 21	2115 ± 20	2112 ± 18	2109 ± 19
**DIC (g/kg)**	1883	1813 ± 43	2009 ± 21	2105 ± 20	2102 ± 18	2099 ± 19
**RE (%)**	**−0.5**	**0.5**	**0.5**	**0.5**	**0.5**	**0.5**
**CO**_**2**_ **(psu)**	10.77	10 ± 1	18 ± 2	31 ± 5	34 ± 3	34 ± 3
**CO**_**2**_ **(g/kg)**	10.82	10 ± 1	18 ± 2	31 ± 5	34 ± 3	34 ± 3
**RE (%)**	**-0.5**	**0.0**	**0.0**	**0.0**	**0.0**	**0.0**
	*n = 1	*n = 63	*n = 24	*n = 22	*n = 18	*n = 15

Concentrations are in μmol/kg; psu = practical salinity unit; g/kg = absolute salinity.

*n = sample size.

It was not evident in this process that the S_A_ is the most suitable scale for calculations of the carbonate system, mainly for speciation and concentration of the carbonate, where the effect of salinity did not generate an error of more than 1% between the different water masses studied, except for the MW, that presented variations above 4% for NTA (n = 1). Regarding Ca^2+^ e B_T_ ’s calculation from the practical and absolute salinity, the RE was between 0.5% and 0% for Ca^2+^ and B_T_, respectively. Although S_A_ represents the better estimation of the salinity, it does not influence the equations that generate Ca^2+,^ B_T_, and TA (Table 7). The use of practical or absolute salinity in the total boron calculation equation resulted in irrelevant differences in this study. However, more verification and experimental confirmation are needed for these water masses, mainly in the MW, where the number of samples was quite small (n = 1). The total average of the B_T_/Cl ratio ((mg/kg)/Cl/‰) for all water masses was 0.223 (for calculated B_T_) and 0.199 (for analyzed B_T_), being similar to that found (0.232) by Uppström [56]. More water collections in the studied are necessary to establish a better empirical relationship between B_T_ and S. Kulinski et al. [57] established this empirical relationship for the calculations of the carbonate system in the Baltic Sea.

The relative error in Table 8 varies between 5.0 and 6.5% for the analyzed and calculated TA values. The relationship between TA and salinity is well known, and several researchers have proposed empirical equations [38, 58, 59]. In this study, the differences between the predicted values and the measured values are somewhat high compared to those presented by Jiang et al. [60], but this may be due to applied equation and small sample size (n<65). New samples were already collected during the dry season and are currently being analyzed. This will allow the development of specific empirical equations for these water masses with smaller differences between predicted and measured values.

**Table 8:** Variations in TA (μmol/kg) were obtained analytically and calculated with the AQM program [1, 13–15]. In calculating the relative error (RE%), the values obtained analytically are the expected values (see item Analytical Procedure: TA and Table 3).

Parameters	Water mass					
	MW	TW	SACW	AAIW	AAIW/UNADW	UNADW
**TA (a)**	2232	2289 ± 44	2219 ± 14	2197 ± 10	2205 ± 10	2213 ± 19
**TA (b)**	2352	2437 ± 10	2355 ± 5	2306 ± 4	2325 ± 4	2332 ± 0.4
**RE (%)**	**5.3**	**6.5**	**6.1**	**5.0**	**5.4**	**5.4**
	*n = 1	*n = 63	*n = 24	*n = 22	*n = 18	*n = 15

(a) = analyzed; (b) = calculated

*n = sample size.

### Inventory and elucidation of physical–chemical processes

**Fig 3:**
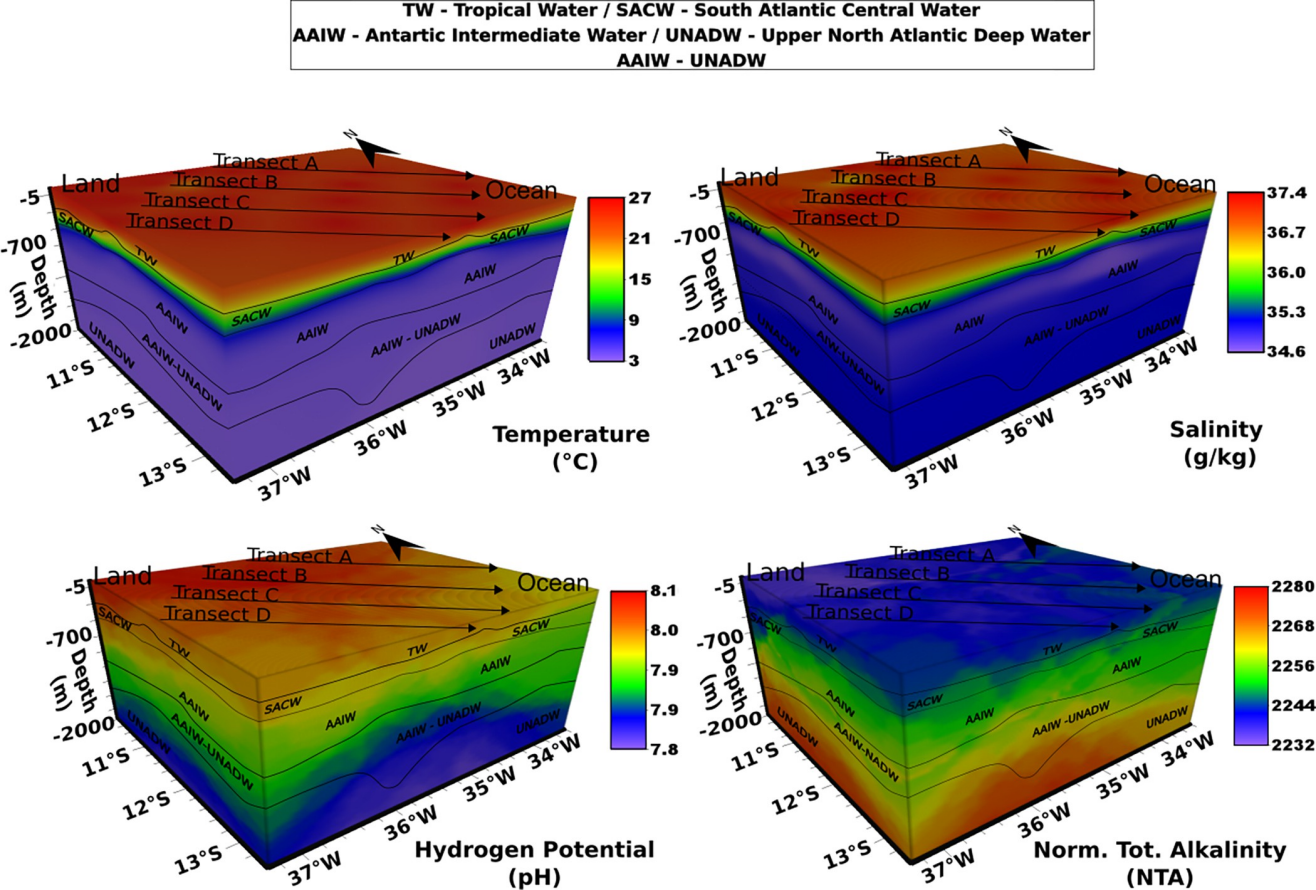
Temperature, Salinity, pH, and Normalized Total Alkalinity (NTA) of the sampling transects, from the internal platform to the abyssal plain.

**Fig 4:**
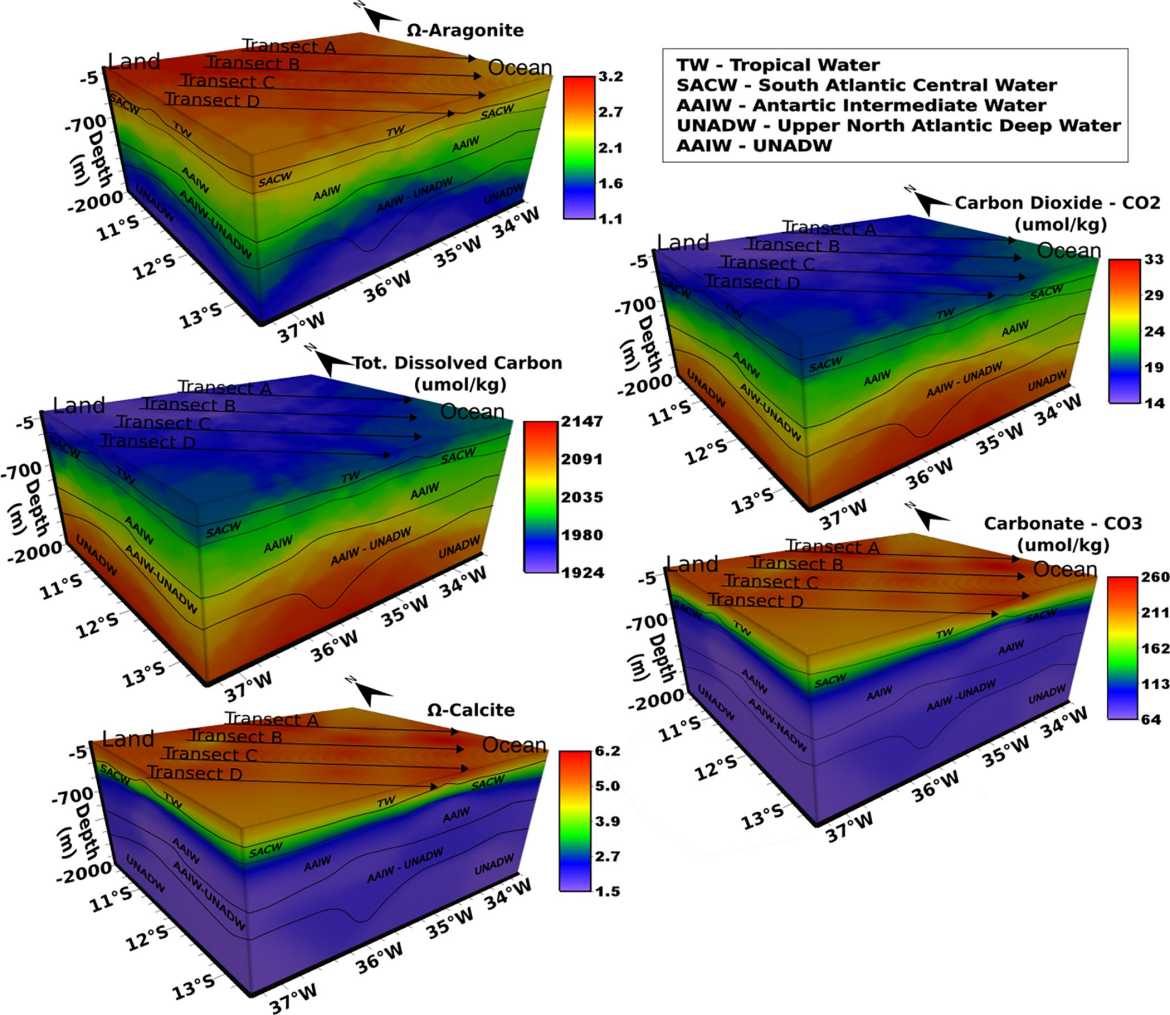
Aragonite saturation state (S-Aragonite), carbon dioxide, total dissolved carbon (DIC), carbonate, and calcite saturation state (S-Calcite) from sampling transects: From the internal platform to the abyssal plain.

**Carbonate system composition and speciation (pH, NTA, and DIC).** The pH and NTA values showed significant variations between the different water masses (coefficient of variation = 1.13% and 1.92%, respectively). In Figs 3 and 4, a drop in pH values can be observed according to the following sequence: TW > SACW > AAIW > AAIW/UNADW < UNADW. The lowest pH value was found in the AAIW/UNADW (7.83) and the highest ones in the TW (8.02). The increase in the content of ${HCO}_{3}^{-}$ and CO_2_ at greater depths may be due to the decomposition of organic matter by respiratory activity [59]. The drop in pH values can be explained by the release of protons from the reaction (Eq 7) between CO₂ and seawater, forming bicarbonate [15] (Fig 3).


$${CO}_{2}+H_{2}O={HCO}_{3}^{-}+H^{+}$$ (7)

In the present study, the water masses had a buffering capacity (ability to keep the pH stable while acids are added) well above the minimum capacity, if considering that TA/DIC = 1. The minimum buffering capacity occurs when the pH value falls close to 7.5 [59], and consequently, the carbonate concentrations are lower.

All pH values presented here were well above 7.8, whereas the NTA concentration of the five water masses showed values of approximately 2200 μmol/kg. Conversely, DIC amounts in distinct water masses showed a 7% coefficient variation, ranging between the minimum of 1845 μmol/kg (TW) and a maximum of 2020 μmol/kg (SACW). Moreover, our data showed raised DIC values for deeper water masses with increasing concentrations of ${HCO}_{3}^{-}$ and CO_2_ (r = 0.95; r = 1.00, respectively; p<0.05), Fig 4.

Average concentrations of ${HCO}_{3}^{-}$ and CO_2_ showed an increase relative to greater depths in the following sequence (values are in μmol/kg):

$$\begin{array}{l} \mathrm{TW}\;({\mathrm{HCO}}_{3}^{‐}=1594;\;{\mathrm{CO}}_{2}=10)<\mathrm{SACW}\;({\mathrm{HCO}}_{3}^{-}=1864;\;{\mathrm{CO}}_{2}=18)<\mathrm{AAIW}\;({\mathrm{HCO}}_{3}^{-}=2037;\;{\mathrm{CO}}_{2}=31)\\ <\mathrm{AAIW}/\mathrm{UNADW}\;({\mathrm{HCO}}_{3}^{-}=2040;\;{\mathrm{CO}}_{2}=34)<\mathrm{UNADW}\;({\mathrm{HCO}}_{3}^{-}=2038;\;{\mathrm{CO}}_{2}=34) \end{array}.$$

The increase in the content of ${HCO}_{3}^{-}$ and CO_2_ at greater depths may be due to the decomposition of organic matter by respiratory activity [61].

**Calcite and aragonite saturation state.** The saturation state of calcite (Ω_Calc_) and aragonite (Ω_Arag_) varied within water masses. The highest values were observed in TW and SACW for both minerals, respectively: Ω_Calc_ = 5.8, Ω_Arag_ = 3.9 and Ω_Calc_ = 3.3, Ω_Arag_ = 2.1. From the AAIW, the values dropped by almost half (Ω_Calc_ = 2.0 and Ω_Arag_ = 1.2). The increase in CO_2_ concentrations is mainly responsible for this scenario since it may raise the carbonic acid content. The following reaction (Eq 3) characterizes the main buffering capacity of seawater, where the consumption of H^+^ and CO_2_ occurs. As the carbonate is consumed from seawater by this reaction (Eq 8), it results in a decreased carbonate saturation state [59].


$${CO}_{2}+{CO}_{3}^{2-}+H_{2}O=2{HCO}_{3}^{-}$$ (8)

Several authors have linked the calcium saturation state (Ω) with the calcification capacity of organisms due to a drop in ${CO}_{3}^{2-}$ availability [62–64], Eq 9. Moreover, previous experiments reported a positive correlation between the calcification rate and Ω, which may lead to an erroneous idea that ${CO}_{3}^{2-}$ governs the calcification rate [65, 66].


$$\Omega =\frac{\left[{Ca}^{2+}][{CO}_{3}^{2-}\right]}{Kps}$$ (9)

Recent research [4, 67, 68] suggested that Ω from seawater does not control the rate of calcification (calcifying fluid). That is, we cannot merely link the availability of carbonate (affected by the decrease in pH values) to the rate of calcification. Notably, to date, no ${CO}_{3}^{2-}$ transporter has been found in calcifying organisms (for example, coccolithophorids). Conversely, there is ample evidence of ${HCO}_{3}^{-}$ transporters. The bicarbonate within the calcifying fluid provides for the formation of calcium carbonate, Eq 10.


$${HCO}_{3}^{-}+{Ca}^{2+}=H^{+}+Ca{CO}_{3}$$ (10)

Reactions involving the formation of calcium carbonate are dependent on the electrochemical gradient (H^+^) between the marine environment and the organisms’ tissue, as well as suitable Ω (higher) in the cytoplasmic fluid. Several gaps still need to be clarified about calcification of marine organisms [69–71].

From the above, pH is a valuable measure by its participation in various chemical equilibrium reactions (proton concentration) [31, 32]. Thus, accurate data on the speciation and quantification of the carbonate system can be obtained at reduced costs [31, 72].

## Conclusions and future perspectives

The present inventory of the carbonate system in the five water masses in the SEAL marine sedimentary basin showed a well-defined scenario among these masses regarding pH, Ω, ${CO}_{3}^{2-}$, DIC, and CO_2_. The variation of these parameters over time remains unknown, requiring seasonal sampling efforts to allow comparisons between water masses. Through a robust and updated database, these parameters can be used to monitor the marine acidification of the studied area at a reduced cost and effort.

More experiments are needed in the studied area to establish more precise empirical relationships between the different parameters of the carbonate system, such as B_T_ vs. S, Ca vs. S and TA vs. S. These relationships may be suitable for future studies of the carbonate system of these and other water masses. This tool may one day replace the laboratory determinations of TA, calcium, and boron since the carbonate system presents slight variation between the calculated and analyzed values, making the whole process more agile and reducing costs.

## Supporting information

S1 Table Sampling stations for the oceanographic cruise in the Sergipe-Alagoas sedimentary basin held in May 2014 on board the Seward Johnson research vessel. (PDF)

S2 Table Dataset for the manuscript "Inventory of water masses and carbonate system from Brazilian’s northeast coast: Monitoring ocean acidification". ID Number = is the identification number of the sample, it is related to the sampling samples. Group = is the number of samples (n) per water mass. psu = is the practical salinity unit; S% = is the absolute salinity; (A) = analyzed values and (C) = calculated values. Latitude and Longitude are the geographical positions (Geographic Coordinates in decimal degrees, Datum SIRGAS 2000).
(XLSX)
