# Supplementary material for: Inventory of water masses and carbonate system from Brazilian’s northeast coast: Monitoring ocean acidification
Source: PLoS One. 2022 Jul 26;17(7):e0271875. doi: 10.1371/journal.pone.0271875 (PMC9321457; doi:10.1371/journal.pone.0271875)
Supplement: S1 Table — (PDF) [file pone.0271875.s001.pdf]

Supplementary material

S1 Table. Sampling stations from the oceanographic cruise at SEAL (Sergipe-Alagoas) sedimentary basin, carried out in May 2014 on board the Research Vessel Seward Johnson.

St #	Latitude (°S)	Longitude (°W)	Depth (m)	Sampling Depths (m)					
				MW[1]	TW[2]	SACW[3]	AAIW[4]	AAIW/UNADW	UNADW[5]
**1**	10.164937	36.024297	24		5				
**2**	10.233732	35.931085	39		5/20				
**3**	10.257768	35.897610	446		5/20	250			
**4**	10.285040	35.859687	1220		5/20	250	750		
**8**	10.564627	35.475918	3344		5/20	250	700/1250	1650	
**10**	10.805872	35.148087	3927		4/20	250	800	1249	1649
**14**	11.448163	34.272912	4595		5/20	202	698	1502	1851
**18**	12.090830	33.397153	4928		6/19	252	706	1260	1667
**19**	12.442687	33.958497	4367		5/20	251	603	1248	1699
**23**	11.781593	34.819570	4396		6/20	252	705	1262	1666
**27**	11.203050	35.573130	3766		5/20	251	800	1250	1598
**29**	11.037793	35.789178	3203		4/20	250	705	1252	1899
**33**	10.746067	36.163010	1320		5/19	255	752		
**35**	10.691080	36.292798	803		4/21	249			
**36**	10.635937	36.324763	556	4	20				
**37**	10.605006	36.349031	115		4/20				
**38**	10.584537	36.374618	43		4				
**39**	11.022943	36.960122	25		5				
**40**	11.062420	36.905630	33		4/19				
**41**	11.123590	36.820557	76		4/20				
**42**	11.142080	36.795572	710		6/20	250	696		
**43**	11.181997	36.740745	1725		5/20	250	749	1400	
**47**	11.421587	36.411888	2944		5/20	251	748	1200	1797
**49**	11.581918	36.192587	3434		5/20	251	601	1298	1749
**53**	12.220437	35.314782	4294		4/20	250	801	1300	1398
**57**	12.860103	34.436907	4412		4/20	251	598	1249	1799
**58**	13.394537	34.953915	4445		4/20	251	650	1350	1800
**62**	12.735687	35.816277	4274		4/20	250	651	1249	1650
**68**	11.911330	36.894945	2890		5/20	299	648	999	1747
**72**	11.705190	37.164422	1664		4/20	251	750	1245	
**73**	11.664335	37.219072	575		5/20	252			
**74**	11.642807	37.248640	73		5/19				
**75**	11.602578	37.299873	35		4				

St#: station identification number; Latitude/Longitude: geographical positions (Geographic Coordinates in decimal degrees, Datum SIRGAS 2000); Depth: location true depths; and Sampling depths per each water mass: [1] Mixture Water; [2] Tropical Water; [3] South Atlantic Central Water; [4] Antarctic Intermediate Water; [5] Upper North Atlantic Deep Water.
